# The Hospital Nursing Supplement

**Published:** 1894-09-15

**Authors:** 


### "The Hospital" Nursing Mirror.

Being the Extra Nursing Supplement of "The Hospital" Newspaper.

[Contributions for this Supplement should be addressed to the Editor, The Hospital, 428, Strand, London, W.C., and should have the word "Nursing" plainly written in left-hand top corner of the envelope.]

### News from the Nursing World.

### ROYAL VISITORS AT BIRMINGHAM.

Glorious weather favoured the crowds who assembled on Saturday in the gaily decorated streets of Birmingham to welcome their Royal Highnesses the Duke and Duchess of York. The foundation-stone of the new building of the General Hospital was certainly laid under most auspicious circumstances, and the first visit of their Royal Highnesses to this great manufacturing centre will long be remembered.

### EASY AND EFFICIENT DISINFECTION.

In Portland Town the recent outbreak of small-pox, the disinfection of rooms after the removal of the patients was, we read, carried out by means of tins of liquid sulphur dioxide. It is, therefore, evident that Dr. Wynter Blyth gives unqualified approval to this simple and efficacious method of fumigating, which has been already mentioned in The Hospital.

### OUT OF TOWN.

"It's quite a treat to see a visitor at this time of year," remarks the Chronic Case, to whom the sight of a fresh face is ever welcome. Life is a very monotonous affair to bed-ridden patients, and "the visitor" is therefore far more essential to them than to the "acute" or "accident" case. As they never go out of town themselves, they must feel it a little bit hard when "everybody" else apparently does so. If kindly visitors would only realise that the duties which they pick up or drop with so much facility are matters of great moment to their humble friends, surely they would be at a little trouble to find substitutes. A vast number of people do not go away at all in the summer, or perhaps only for inconsiderable periods, and therefore the happier mortals who manage to secure a month or two's change should certainly be able to find a substitute to carry on voluntary visiting of infirm and incurables who never go "out of town."

### PROBATIONERS' DUTIES.

Last week, in a provincial journal which has recently proved an efficient champion of the rights of sick paupers, there was an article on "How to Become a Trained Nurse." The writer gave quotations from official papers which contain a certain amount of useful information for aspiring probationers, but some of the interspersed remarks are decidedly misleading. The scrubbing of lockers and doctors' tables which is named amongst the probationers' duties at the London Hospital, for instance, sounds very rough work, whereas, as a matter of fact, these pieces of furniture are generally topped with glazed tiles, and therefore require the lightest of cleaning and no real "scrubbing" at all, and the wooden legs of the tables, and the interiors of the lockers have for years past always been washed by the wardmaids. The writer also says that "on Mondays and Wednesdays they put out the linen for the laundry under the supervision of the sister," which is purely an imaginary picture, as the linen goes daily down the shoot communicating directly with the hospital laundry. It will surprise nurses to learn that "at some hospitals a charge of £13 is made" for the probationer's month on trial, but the names of these expensive institutions do not appear. It seems a pity that an article which should be instructive was not more carefully revised before publication in so popular and widely read a paper. Metropolitan hospital authorities generally prove willing to verify and explain their rules and system of training, and we would venture to suggest to Editors generally the desirability of sending a proof to the authorities mentioned in future in articles like that referred to before publication.

### THE ROYAL BRITISH NURSES' ASSOCIATION.

The fittings and furniture of the new premises of the Royal British Nurses' Association were paid for in part by Princess Christian and in part by the subscriptions of vice-presidents, executive committee, and general council, in response to an appeal made by the Princess. The increased expenditure necessitated by these offices, and also the work of development contemplated by the executive committee require that the society should increase its income. This is to be attempted through the medium of the members of the General Council, who are invited "to collect or subscribe in the form of annual subscription for two or three years as much money as they can towards this increased expenditure." Miss Kenealy has given notice that at the October meeting of the General Council she will move a resolution with regard to the *Nurses' Journal*, the organ of the R.B.N.A.

### ASHFORD COTTAGE HOSPITAL.

An urgent appeal for increased annual subscriptions to the Ashford Cottage Hospital is being made. This little institution is doing very good work, and treated ninety-five patients last year. The present building, erected by Mr. Pomfret Pomfret to the memory of his wife, who was much interested in the hospital, is very pretty and convenient, and it is to be hoped that funds will be forthcoming for the maintenance of an institution which receives patients from fifty-eight parishes.

### THE PAUPERS' SAUCEPAN.

The notes taken by Dr. Fuller, the Local Government Board inspector, after a visit to Bath Workhouse Infirmary, have recently appeared in the local press The condition of the sick wards he found to be generally ill-kept and dirty, the average number of patients being 230, including many acute and bedridden cases; whilst the nursing staff was totally inadequate. A wardsman aged 66 reports that he and the scrubber washed the patients in the acute ward, and "he could get no hot water in his ward except from the copper-house; there is no kettle of hot water. If hot water is got in the ward it is got by using an Australian meat tin as saucepan," the said copper-house being situated on the ground floor. Other existing arrangements call for immediate reforms, but few can present more pathetic possibilities than this ward of acute cases, left during the long night hours without any skilled nursing or hot water. One night nurse has been appointed since Dr. Fuller's visit, and it is to be hoped that this sign of progress may be followed by others in the near future.

### ENGLISH NURSES IN HONG KONG.

The disappearance of the plague from Hong Kong is now reported officially, and the splendid work done by the Europeans during the late terrible epidemic is beyond praise. The English-trained nurses, who have so bravely shared in much extra work and fatigue, have passed well through the ordeal, and their fellow workers in England have good cause to be proud of their friends.

### NURSES AT COBLENTZ.

Coblentz, a city of 30,000 inhabitants, possesses two hospitals, a large one for Roman Catholics containing 450 beds, and a smaller one for Lutherans or other Protestants, containing 45. Both are admirable in their arrangements as regards supreme cleanliness and airiness. Culinary utensils are all brass, and the food is served in white enamelled ware. The beds are very superior—such deliciously soft hair mattresses on easy springs. The large square pillows, too, look comfortable, and the feather quilts on the top give an air of luxury. No German Hospitals are free, they have first, second, and third-class accommodation. In the large hospital at Coblentz five marks a day are charged for the first-class. This secures a separate well-furnished room and includes board, lodging, nursing, and doctor's fees. The second-class pay 3 marks 50 a day, and have also single rooms. The third-class patients inhabit large wards and each pays 1 mark 50 a day. Those who have means pay this for themselves, those who are destitute are paid for by the town. Thus the hospitals are self-supporting and not dependent on voluntary contributions. The prices in the Lutheran Hospital are somewhat less, but the accommodation is not so good. It is hoped that soon a larger hospital will be built outside the town. At the Roman Catholic Hospital the nursing sisters number twenty-seven. The Lutheran Hospital is nursed by Kaisersworth sisters—six in number. Their dress is dark blue, white net caps with frills, tied under the chin. Their aprons also are dark blue with white stripes, turned-down white collars, and no cuffs. On Sundays they are allowed blue woollen gowns and black aprons.

### SPANIARDS AT THE SEA.

The popularity of San Sebastian as a watering-place is somewhat on the decline, although it is again favoured by the presence of the Spanish Court. The little King and his sisters delight in the sands and the sea, and leave their play as happy and dirty as any of the juvenile subjects, who regard the happy trio with much loyal admiration. Probably the decrease in the number of visitors to picturesque San Sebastian this year is due to the superior cheapness of some of the other equally healthy little bays on the coast. Biarritz, which continues to maintain its popularity as a winter health resort, has been almost abandoned by the summer visitors from Spain, who formerly made it so gay. Of course, the great loss in the change of money on the frontier between France and Spain is a drawback which is not without influence.

### STATE REGISTRATION.

In her paper on "The Progress of Nursing," read by Dr. Bezly Thorne, at Buda Pesth, Princess Christian says: "It is the hope of the corporation that the time is not far distant when the State will see the importance of recognising a definite diploma of nursing, and of giving its official sanction to the maintenance of the Register of Trained Nurses." Such a scheme for State registration might be of great value, but would need careful consideration. It would, indeed, be a matter of national importance, altogether distinct from registration by any private or independent body. It has still to be proved whether the kind of women whose preliminary training necessitates numerous examinations before entering as nurse-probationers will not belong more to the eager-eyed, neurotic type of student, rather than to the large-hearted and healthy-minded nurse, who considers science an accessory more than a foundation to her duties of attendance on the sick poor. Princess Christian's great interest in the welfare of nurses makes the paper she has compiled of more than common interest, even to those whom experience convinces them that compulsory registration would be as dangerous as it is unnecessary.

### SHORT ITEMS.

A very successful fête was recently organised at Rushden, in aid of the funds of the District Nursing Association, which is affiliated with the Queen's Jubilee Institute. The entertainment was held on the cricket ground, and was well attended.—The nursing staff of the Royal Hants County Hospital were lately entertained by Mr. and Mrs. Portal, at Malshanger. The prizes gained by the successful probationers at the recent examination were presented on this occasion.—A cricket match between Lightweights and Heavies took place at Leominster, in aid of the fund which is being raised for the erection of a cottage hospital.—The appointment of a permanent night nurse at Oswestry Workhouse Infirmary is under discussion.—The Paris Medical Faculty has 165 women students.—The plans for the new nurses' home at Paisley have been passed. It is to be the gift of Mr. Peter Coats, and will be erected in the grounds of the new infirmary at Egypt Park.—Four nurses belonging to the Devon and Exeter Hospital were upset into the sea as they were returning from a boating trip, but happily they were speedily rescued.—Miss Gibbs (Queen's Nurse), from Addlestone, has been selected Superintendent of the Borough of Portsmouth Association for Nursing the Sick Poor.—A very successful concert took place the other day at Wivelsfield, part of the proceeds being devoted to the Village Nurse Fund.—A branch of the Mester Ross Nursing Association has been formed in the parish of Rosskeen, N.B., by Lady Mackenzie, of Gairloch.—A football competition cup has been provided at Atherstone for the coming season in connection with the Atherstone Nursing Association. Many clubs are expected to enter for the competition.—One hundred and seventeen cases were attended by the District Nurse at Slough during the past year.—A sale of work is being organised for the end of October by the sisters of the Barry, Cadoxton, and District Nursing Association, funds being urgently needed.

### Diet in Disease.

By Mrs. Ernest Hart.

Series II.

### II.—SCURVY AND SCROFULA.

Allied to rickets and resembling it in many of its charcteristics is the once dreaded disease of scurvy. Scurvy is still occasionally seen in hospitals, especially among children, and was once the scourge of the navy, when sailors were fed on salt meat and biscuits. It was, however, discovered that this painful disease was cured by its victims returning to an ordinary mixed diet, and more particularly to the use of fresh vegetables and potatoes. Hence it was argued that scurvy is caused by the lack of the salts and acids contained in vegetables, fruits and potatoes. Strange to say it is not, however, sufficient to give the salts and acids contained in vegetables to prevent scurvy, though in cases when it is impossible to carry even compressed vegetables, owing to their bulk, lime or lemon juice served daily as a ration to sailors commonly will prevent scurvy. It has, in fact, by this means been banished from our navy.

There are some persons who are unwilling to accept the dicta of science and experience, and who put their own uninformed belief before knowledge. The breakdown of the Nares expedition to the North Pole is an example in point, and gave another pitiful illustration, if any were needed, of the cause of scurvy. Commander Nares would not follow the advice of the doctor who argued that it was necessary for the sleighing parties, bound on long journeys across the ice, to take lime juice with them. He considered that dependence on lime juice was a doctor's fad, and held the view that scurvy was caused by darkness, depression, &c., and believed that alcohol would be more useful to the men than lime juice. The party consequently completely broke down with scurvy. The story of the sufferings of the men, as told in a Blue-book Report, is one of the most pitiful ever penned. Many died, and the few remaining managed with great difficulty to reach the ship. They left the spirit casks behind them on the ice-fields, having learnt by bitter experience another well-known dietetic lesson, namely, that in cold and fatigue alcohol is worse than useless, but that under these circumstances hot tea or coffee is the best restorative.

**Symptoms of Scurvy.**—The disease begins gradually. The person who is suffering from scurvy first notices an increasing weakness, constant fatigue, and a sense of soreness in all the limbs. He becomes deeply despondent, and is subject to fits of faintness. Presently the characteristic signs of the disease make their appearance, the gums become swollen, turgid, dark and spongy, swelling up often over the teeth, and sometimes dropping off in gangrenous masses. The teeth are very tender, and often drop out. The breath is particularly foul. Purple patches and bruises appear on the legs; the feet swell, and there is great pain and stiffness in movement. There is often want of appetite, but even if the patient feels inclined for food his teeth are too tender to chew with. A more miserable wretch cannot be conceived than the victim of scurvy; but his cure is certain and rapid if he can only obtain the foods for which his blood is calling, namely, vegetables.

**Dietetic Treatment.**—It is highly satisfactory when one has to treat such a painful and pitiful disease as scurvy, to know that recovery is usually assured by the simple act of changing the diet and putting the patient on plenty of fresh, soft, succulent vegetables, with from four to eight ounces of lime or lemon juice daily. Potatoes and cabbages are the best vegetables. Yams, onions, carrots, turnips, oranges, pears, and apples are also valuable. In extreme weakness beef-tea and milk must be given in considerable quantities until the patient is able to take solid food. When he is able to chew, meat should be given. Under this dietary the symptoms rapidly improve; the swelling and bleeding gums disappear, the teeth become firmer and less tender, the purple patches grow paler and less painful, the tendency to faintness decreases, and the patient grows in strength.

**Scurvy can be prevented** on long sea journeys by each person taking daily at least eight ounces of preserved potatoes, three ounces of other preserved vegetables—carrots, onions, turnips, celery mint, pickles, and three ounces of lime juice. Among recommendations issued by the Board of Trade to shipowners is the following: That each man should have at least two ounces of lime or lemon juice twice a week, to be increased to an ounce daily, if any symptoms of scurvy manifest themselves. By following these simple dietetic instructions scurvy has been banished from our ships, and when it occurs, as in the Nares Polar expedition, it is due to direct neglect of obvious and well-known precautions.

**Scurvy in Children.**—Helpless babies need not have scurvy as they occasionally do if their mothers know how to feed them properly, but owing to the absence of anti-scorbutic elements in their foods, children sometimes suffer from severe and well-marked scurvy. The cachexia, the mental apathy, and depression, the muscular weakness, the purple spots and patches, with deep extravasations of blood, the tenderness of the limbs, the swollen ankles are present, and the most characteristic symptom of all, a soft, livid, purple and spongy condition of the gums, which are sometimes so swollen as to hide the teeth altogether and protrude from the lips in lobulated bleeding and ulcerated masses. Unless they be relieved death occurs from syncope, or from increasing weakness. Dr. Cheadle gives among others a case typical of the cause and treatment of scurvy in an infant. A healthy child, whose parents were in good circumstances, was suckled till it was six months old; it was then weaned and fed entirely on oatmeal and rusks mixed with water and no milk; condensed milk, which had been previously tried, being thought to disagree. At ten months mutton broth was added. This diet was continued without change till the sixteenth month. It will be remarked that this diet was deficient in animal fat, and contained little nitrogenous material. It was a diet likely to develop both rickets and scurvy. Most children of a year old are given milk and potatoes. In spite of the administration of potatoes and cod-liver oil well-marked scurvy developed in this case. The treatment consisted of pure milk, fine potato gruel, and raw meat; in a few months the child was running about strong and well. Numerous other cases might be quoted. There is a wearisome similitude in all of them. The little patients are nearly always bottle-fed children under two years old. "In no instance," says Dr. Cheadle, "have I seen the disease arise in an infant at the breast, or when fed on an ample supply of good cow's milk. Oatmeal and water, bread and water, various patent farinaceous and dessicated foods, peptonised condensed milk, sterilised milk, pancreatised food and milk, German sausages, bread and butter and tea, beef-tea, gravy and bread, in cases with no fresh milk at all, in a few with a very small amount only, are the dietaries on which I have seen scurvy develop. And in these cases with children, as with adults, the improvement which immediately follows the administration of anti-scorbutics is one of the most remarkable facts in the whole range of medicine, and a convincing proof of the condition being true scurvy."

### SCROFULA.

This is a disease which is supposed to be hereditary, and which is allied to consumption. A scrofulous child does not, however, often become consumptive. The signs of the disease are well known. The child is thin, pale, and unwholesome-looking; it has not the gaiety of childhood; and the glands, especially those of the neck, become hardened and swollen, and often slowly suppurate and discharge a purulent cheesy substance. The edges of these abscesses are ragged, and, when healing takes place, depressed puckered cicatrices are formed. These are unsightly, and are characteristic of a scrofulous diathesis. Malodorous discharges from the ears or nose are not infrequent in scrofula.

**The Treatment** is simply nourishing food and fresh air. Care should first be taken to ascertain what the child can digest well, and all indigestible food should be avoided. "An abundant supply of good milk should be the basis of its diet; also wholemeal bread and plenty of butter." Yet fatty foods are what seem to be needed in scrofula, and as puddings are, as a rule, better liked by children than meat, there is little diffidifficulty in getting them to eat food so agreeable to them. A plentiful supply of butter and cream with breakfast and tea, bread and dripping, suet-pudding with jam and treacle, apple and suet pudding are all good foods for the scrofulous. Codliver oil is the doctor's sheet-anchor, but where this is not well borne, or there is a dislike to it, cream may take its place. To make this digestible it is only necessary to add a teaspoonful of cherry brandy to a wineglassful of cream. Cream and soda-water will be found an excellent drink for the scrofulous. The clothing should be of wool, and every opportunity taken to give the child a healthy active life in the sunshine or by the seaside. The dreaded disease may thus be warded off or ameliorated, and the weak and scrofulous child grow into a healthy adult.

### A Home for the Dying.

We have received a pleasant little word picture of the Hospice for the Dying at Dublin, visited lately by a correspondent. Very bright were the wards, bright the faces of the kindly nuns, and the whole place cheery and clean. Truly a veritable asylum for the poor creatures who seek the shelter which the hospice affords to a class which no other institution will shelter. Occasionally, happily, the verdict is a mistaken one, and the nuns lose their patients through convalescence and not through death. The hospice contains 100 beds, tended by 20 to 30 nuns. There are two wards for consumptives. Those who are well enough amongst the patients at the hospice attend service in the beautiful chapel, made more beautiful still by adorning flowers. Those who do not hold the same faith as the good nuns are attended by their own minister, for Protestants as well as Catholics are welcomed to the hospice.

### A Case for Assistance.

Miss Bell and Miss Farrow desire most gratefully to thank all contributors to this case, and announce that they have now received more than the sum asked for. A statement of accounts and particulars of what has been done with the money will be published later. The following subscriptions have been received up to the present date: Doctor, Matron, and three Nurses (Shrewsbury), 10s.; Sister-in-charge (Newton), 2s. 6d.; Nurses H. M., 1s.; A. M. H., 1s.; H. L., 1s.; A Reader, 20s.; Ex.-M. (Tunbridge), 2s. 6d.; Fellow Worker (Alnwick), 2s. 6d.; Nurse (Penzance), 2s.; Sister Olive, 3s.; Lady Patient (Kew), 2s. 6d.; Nurses Ada, Hilda, Amy, Bessie (second collection), 5s.; Mr. S. C., 5s.; Nurse E. (Penrith), 10s.; M. A. M., 3s.; Matron and Nurses, Tamworth Hospital, 25s.; Two Hospital Readers, 1s. 6d.; A Paris Nurse (5 frs.), 3s. 11d.; The Nurses, Beckett Hospital, 5s. 6d.; Nurse Cade, 2s. 6d.; A. L. P. (Honiton), 5s.; H. L. W. (Malvern), third donation, 5s; Doctor and Staff, Kingston Isolation Hospital, 25s.; Mrs. P. and Two Nurses, 7s.; C. H. W., 8s. 6d.; Lucy S. (Rochdale), 1s.; Nurse Hemming, 2s. 6d.; E. K. H. (Benson Home), 3s.; M F, 20s.; F. A. B., 31s. 7d.; the Nursing Staff of the Birmingham Infirmary, £3 15s.; making a total of £27 14s.

### Nursing in Germany.

By a German Correspondent.

Two cases of genuine leprosy have been discovered in the clinical department of the University of Breslau. A few days ago a workman, coming from the neighbourhood of Memel, died of this terrible disease, and another man, belonging to the same district, is now under treatment. The authorities have taken the most careful measures of isolation.

It is somewhat of a novelty to find an article on nursing contributed by a theologian at the special request of an editor. Yet Dr. F. Zimmer, of Herborn, Professor of Divinity, has recently penned a long and able paper on "How to obtain Lady Nurses," and a second is to follow in the next issue of the "Zeitschrift für Krankenpflege." Proceeding from a Lutheran, it is yet singularly marked by broadmindedness and absence of prejudices. Some weeks ago I referred to Dr. Brückner's book on this same subject. Since then Frau Mathilde Weber, the wife of a professor of medicine, has written "Why are we in want of Deaconesses and Nurses?" Much has already been accomplished this year towards supplying the need in future. A deacon's seminary for the training of male nurses is in course of erection at Elberfeld (though, the writer of this article particularly emphasises nursing as essentially women's work). A "Tochterheim" has also been opened at Cassel, in which women belonging to the better classes are to be trained for nursing. These plans are in the hands of an excellent committee, composed, amongst others, of eminent medical professors, the director of an ecclesiastical seminary, and a professor of theology.

In his article Dr. Zimmer points out the advantages which doctors, patients, and also the friends of patients, and the hospitals themselves would reap from the appointment of educated nurses, not attached to any special "order," as the latter are liable to be summoned from their duties at any time to fill offices in the "Mutterhaus," strangers being appointed in their place. Probably some who look after the bodily comforts of those under their care are devoid of sympathy, or are unfit to command the respect which would be obtained by a lady filling the same position.

Judging from the last census in the German Empire, 41 per cent. of the female population between the ages of 18 and 50 are unmarried or widowed, and it is calculated that on an average only four out of ten single women of the middle and higher classes stand any chance of marriage. Why, then, do not these women, who doubtless are willing to devote their services to some good cause, take up the profession of sick nurses? How often the relations of some poor creature in dire need of skilled care apply in vain for a sister to share the weary hours of watching with the tired mother or wife! Regret is expressed that no help can be given, "as there is no nurse at liberty." What a busy life a sister's is, too. She must not only become proficient in the art of nursing, but is often called upon to undertake such duties as washing, serving, &c., in her turn; in fact, she often fulfils in Germany the duties of a maid, without any of the pleasures. On account of the restrictions laid upon nurses, many who would willingly proffer their services prefer to take a situation as "Stütze der Hausfrau," by no means an enviable post, except that a fair amount of liberty is permitted. Dr. Zimmer attributes this preference to the supposition that in such a capacity they are more likely to be sought in marriage. This view of the matter is certainly derogatory to the dignity of German women. It is true the latter lack the independence and self-confidence of their English sisters, and are infinitely more impressionable; but when the position of nurses is improved in this country, I am confident there will be no need to appeal for competent workers. I have myself been nursed by Germans, and can testify to their skill, kindness, and ready sympathy.

### Nursing on the Labrador.

(By a Correspondent.)

"There is no fish yet," is a remark of which the full significance can be fully realised when heard on the lips of the fishermen of the Labrador. The cod, on whose coming their entire livelihood depends, lingered late this season, and men and women talked very gravely about it to the sympathising listeners who arrived in the "Albert," sent out by the Mission to the Deep Sea Fishermen.

In England where fruit and vegetables come within the ken of most people, it is difficult to realise a condition of life in which "the fish" forms the only available provision.

Yet a short stay amongst the fishers suffices to make English nurses share the interest in this absorbing topic, and they grow conversant with the methods of the fishing industry.

When one doctor and nurse had been installed at Battle Harbour at the little hospital which did so much good service last year, the good ship "Albert" sailed off for Indian Harbour on July 13th, the other doctor and nurse hoping soon to reach the hospital there. Last year this hospital was in process of building, and many suggestions had been made and plans laid down for its completion, and naturally some curiosity prevailed as to whether everything would be found ready. However, it was one thing to start off for Indian Harbour and quite another to get there. On the morning after leaving Battle Harbour an ice field and a coming fog proved serious obtacles to progress. However, as on the previous evening, a glorious sunset was witnessed by the voyagers, and the sunset glow was most lovely at the horizon, stretching far north to a deep crimson belt, shading into bright crimson, and then fading away into the palest pink and yellow. The sky was of a deep blue, and the sea like glass, whilst the moon, nearly full, cast a silvery track upon the ocean, and this track was crossed every now and then by large pieces of snow-white ice floating lazily southwards. The low moaning noise of the ice-field was the only sound on that still and lovely night.

Next morning there was a fog with rain, and the ship was soon fighting her way through the ice, sometimes getting stuck fast for a time, but eventually the ice gave place to her. The glare of the sun on the dazzling whiteness made eyes ache, but it was very lovely to see the great pieces of ice, all shades of blue amongst the pure white, and all floating on a sapphire sea. The shape of the bergs was very odd, one, for instance, exactly like a mushroom, stood on a flat piece of ice, and beside it the stem of another, the round head of which lay just beside it.

Two immense whales passed by, they were quite as long as the ship, and a number of blow fish almost surrounded the "Albert," and then two seals came in sight. The ship got as far as the Ganet Isles only to find there a perfect block, and as the wind had changed the captain decided to get back to Gready if possible—a distance of about ten miles. There were many schooners lying in that harbour, and also in Cartwright, which were unable to get north. The "Albert" got on very slowly, and presently a number of boats went to her assistance, and finally towed her into the harbour. The doctor busied himself all the morning in seeing the patients, who always appeared in large numbers on the hospital ship's arrival.

The diet of the young babies seems to be as varied as amongst the poor in England, for one mother mentioned as desirable for a sick infant, only four days old, nutmegs, magnesia, gin, or wine!

A visit had to be paid to a sick man at Mullin's Cove, a place which took two hours' hard rowing to reach. He was very grateful for the medical comforts supplied to him. The doctor saw 94 patients next day, but thick fog and a wind which kept the ice near the shore combined to hinder attempts to reach Indian Harbour. On the 19th a clear sky and a fresh breeze proved favourable, and the ship passed safely through the ice from "Tumble-down Dick" to "George's Island." Much snow was still lying on the hills, many of the islands having a fringe of ice around them. It had certainly been a very hard and long winter for the poor folks on these barren coasts. When the ship at last drew near to Indian Harbour a group of people was visible on the high hills above Payne's Cove, and directly the ship arrived opposite to them they all fired off their guns. This was the signal to those farther up the harbour, so they fired too, and from every schooner in the bay the same welcome was given to the "Albert."

After being hospitably entertained at tea, the doctor and nurse were taken up to see the hospital they had come so far to serve. English readers must not let their fancy run away with them, picturing an ideal "cottage" or even a "temporary" hospital, for neither of these is like the reality. To begin with, this little erection had neither windows nor chimneys, for nobody at Indian Harbour was capable of building the latter, and therefore it was decided to use iron pipes for the purpose, the stoves being at the end of the rooms. The two largest of these in front form the wards, and will hold eight beds and a cot each; and downstairs there is a sitting-room and a kitchen, and a nice long room for meetings and for a waiting-room for patients. At the end of this there is a small dispensary. Much work still remained to be done, tables and chairs to be made, shelves to be put up, and a door or two moved or readjusted. So the crew set to work, and everybody was soon very busy. Considerable difficulty was experienced in getting a girl to act as servant in the hospital. Even the woman who undertook to do the washing warned the nurse that if much fish was caught she might have to give up her laundry work. Three girls were eventually lent by someone for a day to scrub the top rooms, but they were very slow workers, and did not achieve between them as much as any ordinary English charwoman would have accomplished in half the time. From the ship, where all the party slept during this time, a most disturbing sight was witnessed on Saturday, July 28th. The Captain called out, "Doctor, your hospital is on fire!" and smoke was to be seen rising from one end of the new building. Crews from the schooners promptly assisted the men from the "Albert" in extinguishing the flames, and men and women from far and near came running up and carried water. One brave fellow who had helped to build the hospital seems to have first noticed the smoke issuing from the sitting-room, and he seized a bucket of water, and bursting open the door flung the water on the burning wood. He also cut away a tarpaulin, which was covered thick with tar, and thus saved the roof. The fire arose from an over-heated beehive stove. Most of the rooms were flooded, of course, and what with burnt wood and water the hospital, which had been made so clean the night before, even the beds having been aired, was converted into a very miserable looking place. The same day a patient arrived, too ill to be sent away. So nursing had to be combined with cleaning and furnishing, food being cooked and brought from the ship pending the arrival of stores. It is evident that anyone who undertakes nursing on the Labrador must be willing and able to turn her hand to a great many things hardly included in ordinary "training," and yet all of them bearing immediately on the welfare of the sick.

### Everybody's Opinion.

A WARNING.

"Experimentia Docet" writes: May I, through the columns of your widely-read paper, warn the members of our profession, especially the matrons of institutions, against an impostress? She purports to be a probationer from the Work, house Infirmary, Cardiff, up for a day's holiday, and says she has had her pocket picked and has not enough coin to wire to the matron, and does not know what to do. When she visited me I took down the address of the matron and said if I got a satisfactory reply I would advance the money for her journey. I then gave her a letter to the matron of a Nurses' Hotel, undertaking to pay for two nights' lodging. I now learn that the name she gave me is not known at the infirmary, and on inquiry at the Nurses' Hotel I find she has never been there. The woman was clever enough to use the one appeal women who work never dare turn a deaf ear to-namely, she "had nowhere to spend the night." She is young, rather good looking, and with a silly sort of manner.

TRAINING FOR MALE NURSES.

A "Male Nurse" writes: I was not only surprised but pleased to see your article referring to male nurses for England. I trust something may soon be done by way of establishing a centre where men might be trained for nursing. Would you make a few suggestions, sir? I think the only thing needed is for someone to suggest, and a beginning would soon be made. I left very good prospects in life purposely to become a nurse, and for some years now I have acted as nurse-attendant to private individuals, and, I can safely say, with marked success. I have always found the physicians in attendance very kind in recommending me fresh cases. I attribute my success entirely to the fact, I liked the work. It is as you say a grievous thing that no instruction is available for men who have the aptitude and liking for the art of nursing. Hoping the matter will not rest with your article of last week.

### The Nurse Dolls.

A Grand Garden Fête in aid of the funds of the South Oxon Benefit Nursing Association was recently held at Thame Park by the kind permission of Mr. W. A. Wykeham-Musgrave. Many novel entertainments took place in the grounds, and The Hospital collection of Nurse Dolls was exhibited in a tent, receiving there much attention from the crowds of visitors present. The hon. secretary, Miss Holloway, is to be congratulated on the admirable arrangements made by herself and the two energetic committees.

### Notes and Queries.

**Queries.**

(138) *South Africa.*—Please tell me where I can get information respecting nursing in South Africa?—*E. C.*

(139) *Training.*—Advice wanted.—*A. P.*

(140) *Medico-Psychological.*—Some information wanted as to training for the examination of this association.—*Mental.*

(141) *Hospitals.*—Where can I get names of hospitals abroad, or agencies connected with them?—*A. A. F.*

(142) *Monthly Nurse.*—What is the best way of getting employment?—*E. H. and Probationer.*

**Answers.**

(138) *South Africa* (*E. C.*)—The Hon. Secretary of the Cape General Mission is Miss Everett, 8, The Paragon, Streatham Hill, London. See The Hospital of September 16th, 1893, p. ccxlv.

(139) *Training* (*A. P.*)—We should advise you to apply to the Hon. Secretary of the Workhouse Infirmary Nursing Association, 6, Adam Street, Strand, London.

(140) *Medico-Psychological* (*Mental*).—You had better write to the Hon. Secretary, Dr. Spence, Burntwood Asylum, Lichfield.

(141) *Hospitals* (*A. A. F.*)—You should write direct to the matrons of the hospitals whose names you will find in "Burdett's Annual," published by the Scientific Press.

(142) *Monthly Nurse.*—Write to the Hon. Secretary of the Midwives' Institute, enclosing stamped envelope for reply. Address, 12, Buckingham Street, Strand, London.

*Mr. James Pond.*—We think you must be under a misapprehension, as no such arrangement could possibly be entertained.

### Nursing in South Africa.

An English nurse of fifteen years' experience, who is a member of the Royal National Pension Fund and also of the R.B.N.A., sends us the following discouraging account of the prospects of an English nurse in South Africa. If there is a better side, will some other correspondent send it for publication?—Ed., *T. H.*

As trained nurses in England are having their attention called to South Africa as a new and wide field for work, I think perhaps a little information as to the conditions under which such work is done may be of value. For nurses wishing to obtain work in hospitals, and already trained in England, there is absolutely no opening. The only large hospital in the colony is the New Somerset, Cape Town. The matron is a sister belonging to the Sisterhood of All Saints. There are no nurses on the staff who have been trained out of the hospital, as the Sister prefers colonial-born girls, and to train them herself. They are generally the daughters of small storekeepers or farmers. Kimberley—the next in size, but more of a paying patient hospital—takes much the same class, with a few delicate English girls thrown in, who are quite unfitted for the work, and are always knocking up, which makes the work very hard for the few strong ones. There are about 40 nurses and 200 patients. The patients pay 5s., 7s. 6d., 10s. 6d., and 15s. per day. The nurses' salaries are £30, £40, and £50, without uniform, and as everything is three times the price it is at home, it is a difficult matter to make both ends meet.

All the other hospitals, with the exception of the Albany in Grahamstown, and the Port Elizabeth Government Hospital, are of the Cottage class, having generally a charge nurse, matron, and two assistant nurses. As a rule, they are miserable places, undernursed, badly constructed, with no sanitary arrangements or quarters for nurses. The doctor, as a rule, is young and inexperienced, knowing nothing of the requirements of a hospital or of its management. The greatest difficulty in these small hospitals is servants. You must employ coloured labour, and no one who has not lived in the country can have the faintest idea of their dirt and general incapacity. Private nursing all over the colony is very trying. There are such a number of nurses and so little work, unless you will take monthly, and, as usual, all over the world that branch is never idle.

Most of the houses in which you nurse are wretched, small rooms, no linen, badly cooked food, 18 hours on duty, and then to get your rest in the bed occupied by some one else; a regular Box and Cox affair. The way the average colonial herds together is something wonderful—five and six people in a bedroom, all ages and sexes. A married girl of 18, whose husband is up country, and she expecting her confinement, sharing her bedroom with younger brothers and sisters. Women in the colony have no sense of decency. The tone even among the best class is frightfully low and sensual. Private nurses get £2 2s. per week, and when out of work cannot live for less then 25s. per week even in Cape Town, the cheapest town in the colony.

The climate of Cape Town is much the same as England: cold, wet winters. Up country the winters are dry and cold, hot, and rain storms in the summer. This is a true and not over bright account of nursing in South Africa. I hope nurses will think twice before they throw up anything certain in the old country for the doubtful chance of work out here. My advice is, think well before you leap, truly into the dark. C. C. B.

### Wants and Workers.

*A Home for an Epileptic Boy.*—Can any reader give information where a boy of ten years old suffering occasionally from epileptic fits could be received into a cottage home? Could pay 10s. a week.—Secretary, Trained Nurses' Club, 12, Buckingham Street, Strand, W.C.

### Our American Letter.

NEWS comes from Boston to the effect that the new hospital for contagious diseases at Chester Park will be ready for occupation by the end of the year. Mrs. Flentje, who held the post of superintendent of the German Hospital Training School, N.Y., for some years, has recently been appointed superintendent of the nurse training department connected with Dr. Watkins' Sanatorium for Women's Diseases, Montgomery, Alas.

Better nursing for the insane is urgently demanded in many countries, and Dr. Weir Mitchell brings the subject home to us in his recent reports of the condition of the asylums in some thirty cities where good nursing is non-existent. All thoughtful physicians agree with Dr. Weir Mitchell's estimate of the supreme importance of skilled attendants for patients suffering from diseases of the brain.

After October all graduates at the Carney Hospital, South Boston, will be obliged to undergo three years' training instead of the two years' course which has hitherto been the maximum. Miss Adelaide Nutting, acting superintendent of the Johns Hopkins Hospital Training School, gives a most encouraging account of the progress of the Alumnæ Association, which was founded there only two years ago, and now numbers 76 members. Financial aid is secured in sickness by means of the benefit fund which is managed by an executive committee, and another equally important fund aims at securing rest for weary members. It is hoped that in time this latter fund will be sufficient to provide a suitable and comfortable home during periods of varying length for all members requiring rest and unable to go long journeys to their families. Pleasant homes for private, and central ones for district nurses, are also included in the plans of usefulness to which the Alumnæ Association at Baltimore desires to direct the attention of the graduates.

The Alumnæ Association connected with the Illinois Training School has drawn up some excellent articles, commencing with "Loyalty." "It is the first duty of a nurse to be loyal to the school from which she was graduated. If a nurse remains long enough in a school to accept its diploma, she owes it her allegiance, and should avoid adverse criticism on its management. As an expression of loyalty, a nurse should always wear the complete uniform of the school when on duty."

The *Trained Nurse* for this month contains a very appreciative notice of the late Miss Eliza Perkins, whose life of "simplicity and Godly sincerity" won for her universal respect and affection during the whole course of it. At the age of 62 she became superintendent of the Belle Vue Training School, "which had been struggling for existence for two years." Miss Perkins' success during the 15 years she held this post in a hospital with 600 patients and a training school with sixty pupils is well known. She died on May 28th.

### The Book World for Women and Nurses.

[We invite Correspondence, Criticism, Enquiries, and Notes on Books likely to interest Women and Nurses. Address, Editor, THE HOSPITAL (Nurses' Book World), 428, Strand, W.C.]

### MAGAZINES OF THE MONTH.

Two articles in the WESTMINSTER REVIEW for the current month deal with subjects poetical. In "The Forerunner of Keats," Miss Alice Law touches up our memories of Henry Kirk White, a direction in which it is as well the reading public should be touched up. Of his meteor-like existence we know, alas, so little—less, perhaps, too, are we acquainted with his verse. The advent of Keats, of Keats who was so like him, sounded the young poet's death-knell. To-day, it is only too truly, as Miss Law puts it, that "It is only among the rusty unearthed treasures of some second-hand market bookstall that we seek his too unvalued book." A note on the occasion of the anniversary of Burns, by D. F. Hannigan, is delightfully written. All the articles in the current issue of the "Westminster Review" repay one for the reading. "What Evolution Teaches Us" and "Co-operation and the Agricultural Depression" deal on much debated grounds of political economy and science respectively.

The pages of the PALL MALL MAGAZINE open with a short story by Mr. Clark Russell, the plot of which, of course, is laid at sea. "So Unnecessary" is written in the author's happiest vein, though we find it far too short. Lord Roberts commences "The Rise of Wellington" in this number. Mr. Walter Besant contributes an illustrated account of "The History of Westminster."

Subjects of a deep human interest find discussion, as usual, in the pages of THE HUMANITARIAN. Mr. Grant Allen's views on "The New Hedonism" are demonstrated in a strong, spirited manner. "Pawnbroking" is described by George Washington Moon in an entertaining, if pathetic, chapter.

CASSELL'S FAMILY MAGAZINE presents a uniformity of interesting subjects for our perusal this month, "Sun Spots," from the pen of that prolific writer Sir Robert Ball, and "The Identification of Criminals" being the chief features in the pages. In a lighter vein is "A Little Misunderstanding," a short story, brightly and cleverly written by Bradford Whiting.

"IN DUE SEASON." By ALICE GOLDWIN. (London: Digby, Long, and Co., 1894.)

"In Due Season" is a book which more especially will make its appeal to the nursing world. Indeed, although we are not definitely so informed, we can make a fairly shrewd guess that the volume is an outcome of the writer's own experience as a nurse herself. There is a certain ring of reality pervading the pages of Miss Alice Goldwin's book, and the facts and circumstances therein detailed seem to come from a personal standpoint. It tells us the story of a woman's life—of a chequered career through the devious paths of poverty and want. We are introduced to Alice Evans first as a journalist, then as a hospital nurse. And around her later experiences it is, that the chief interest of the story centres. In fact the first few chapters leading up to this are rather tedious reading, the minutiæ and accessories of the tale being too laboriously entered into. But the heroine sustains the reader's interest to the end. There are several other characters in the book, equally well and consistently depicted, among whom Dr. Arkwell, the leading physician in a big northern town, stands out conspicuously. With him are our sympathies from the beginning. Hampered early in life with a querulous doll-like wife, the doctor's success and his brilliant career are handicapped in no inconsiderable a manner. From their first meeting Dr. Arkwell and Alice Evans are much struck with each other, and the title of the book anticipates the happy ending, for it happens that after a time the frivolous wife dies, and "In Due Season" we leave our heroine the proud partner of the illustrious doctor. The book, as we have already said, will find many readers, especially amongst the nursing world, which life it reflects in a trustworthy conscientious manner.

